# Editorial: Methods and application in experimental pharmacology and drug discovery: 2021

**DOI:** 10.3389/fphar.2022.1097770

**Published:** 2022-12-14

**Authors:** Letizia Polito, Wawaimuli Arozal, Yuhei Nishimura, Aprilita Rina Yanti Eff, Chiranjib Chakraborty

**Affiliations:** ^1^ Department of Experimental Diagnostic and Specialty Medicine—DIMES, General Pathology Section, Alma Mater Studiorum—University of Bologna, Bologna, Italy; ^2^ Department of Pharmacology and Therapeutics, Faculty of Medicine, Universitas Indonesia, Jakarta, Indonesia; ^3^ Department of Integrative Pharmacology, Mie University Graduate School of Medicine, Tsu, Japan; ^4^ Department of Pharmacy Faculty of Health Science, Esa Unggul University, Jakarta, Indonesia; ^5^ Department of Biotechnology, School of Life Science and Biotechnology, Adamas University, Kolkata, West Bengal, India

**Keywords:** experimental pharmacology, drug discovery, methodology, validation, experimental models

Searching for new drugs is an expensive and time-consuming process, but it remains vital for the treatment of many old and new diseases. The availability of current advanced technologies has led to an acceleration of the drug discovery process, facilitating further development of personalized therapies. Sometimes, to obtain the desired therapeutic goals, the drug administration strategy can be of primary importance. Equally important, however, is the understanding of the complex mechanisms of action of new drugs on different cellular and molecular targets, both when the drugs are used alone and when they are administered in combination with other molecules.

This Research Topic was conceived with the aim of giving an update on the latest experimental techniques and methods used to investigate fundamental questions in experimental pharmacology and drug discovery research, with a focus on methods used to shed light on the mechanisms of drug action. This Research Topic includes: two original research articles focused on osteoclast involvement in pathological conditions; two review articles, one focused on the role of FUNDC1 in mitophagy in cardiovascular diseases, and one focused on the use of anti-PD-L1 antibodies as carriers for toxic payload delivery; and two methodological articles, one focused on EndoMT modulation, and one focused on generating neuron-like cells with glutamatergic phenotype.

Total joint arthroplasty can cause local inflammation, osteoclast activation, and periprosthetic osteolysis. So far, the pharmacological strategies targeting osteoclasts have not been fruitful. Huang et al. found that Lonafarnib inhibits farnesyltransferase, suppressing the ERK signaling pathway and preventing osteoclastogenesis in titanium particle-induced osteolysis. The influence of Lonafarnib on osteolysis prevention *in vivo* was demonstrated utilizing a titanium particle-induced mouse calvarial osteolysis model.

Also, the inhibition of osteoclast differentiation and maturation has generated mainstream research interest in the prevention of osteoporosis. Isoliensinine (Iso) is a dibenzyl isoquinoline alkaloid with antioxidant, anti-inflammatory, and anti-cancer properties. Liu et al. investigated whether Iso might play an anti-osteoporosis role by suppressing the differentiation of osteoclasts *in vitro* and *in vivo*. They found that Iso inhibits the formation of mature multinuclear osteoclasts induced by RANKL, reduces bone resorption, and prevents osteoclast-specific gene expression by blocking the nuclear translocation of NF-κB p65. They also found that the effects were dosage-dependent. Iso attenuated bone loss in an osteoporosis animal model (ovariectomized mice) and significantly promoted BS, Conn. DN, Tb.Th, TB.N, and BV/TV Index.

Mitophagy is a form of autophagy that selectively degrades damaged mitochondria involved in the molecular mechanism responsible for several diseases. FUN14 domain containing 1 (FUNDC1) is a mitochondrial receptor located in the outer mitochondria membrane that governs the mitophagy process. Mao et al. explored the role of FUNDC1 in the occurrence, progression, and prognosis of cardiovascular diseases, indicating a novel role for this mitophagy receptor in the regulation of mitochondrial homeostasis in the cardiovascular system.

Programmed cell death protein-1 (PD-1) and its natural ligand programmed cell death ligand-1 (PD-L1) form the PD-L1/PD-1 axis, a well-known immune checkpoint mechanism, which is considered an interesting target in cancer immunotherapy. To date, three anti-PD-L1 antibodies have been approved by the FDA, namely, atezolizumab, durvalumab, and avelumab. Despite the good results reported in clinical trials with anti-PD-L1 antibodies, a significant number of patients do not respond to the therapy. Zanello et al. examined the literature regarding PD-L1 targeting antibodies utilized as carriers for toxic payloads (toxins, drugs, enzymes, radionuclides, *etc.*) to form immunoconjugates for the potential elimination of neoplastic cells expressing PD-L1. The real potential of anti-PD-L1 antibodies as carriers for toxic payload delivery is considered and extensively discussed.

Endothelial-to-mesenchymal transition (EndoMT) has been described in different pathological conditions, including organ/tissue fibrosis. The modulation of the EndoMT process could have therapeutic potential in many fibrotic diseases. Krishnamoorthi et al. developed an *in vitro* method to induce EndoMT with Nω-nitro-L-arginine methyl ester hydrochloride (L-NAME) and angiotensin II (Ang II) followed by a protocol to study the reversibility of EndoMT. This method can be useful as a model to screen and identify potential pharmacological molecules to target and regulate the EndoMT process, with applications in drug discovery for human diseases.

The human SH-SY5Y neuroblastoma cell line is widely used in neuroscience research as a neuronal cell model. Following differentiation to a neuron-like state, SH-SY5Y cells become more morphologically similar to neurons and form functional synapses. Martin et al. developed a novel method for generating glutamatergic SH-SY5Y neuron-like cells utilizing B-27, a supplement commonly used in neuronal culture. The authors demonstrate that B-27 can support the production and survival of large numbers of differentiated SH-SY5Y cells with a glutamatergic phenotype, thus opening new opportunities for research into the role of glutamatergic signaling in the biology of neurological disorders.

In conclusion, the published articles for this Research Topic cover numerous aspects of the ongoing research into the methods and applications of experimental pharmacology and drug discovery. It is hoped that this collection of articles will provide readers with a useful update on the latest experimental techniques and methods used to investigate fundamental questions in experimental pharmacology and drug discovery research.

